# Challenges in biobank governance in Sub-Saharan Africa

**DOI:** 10.1186/1472-6939-14-35

**Published:** 2013-09-11

**Authors:** Ciara Staunton, Keymanthri Moodley

**Affiliations:** 1 Centre for Medical Ethics and Law, Department of Medicine, Faculty of Health Sciences, Stellenbosch University, Stellenbosch, South Africa

## Abstract

**Background:**

Biological sample and data transfer within and out of Africa is steeped in controversy With the H3Africa project now aiming to establish biobanks in Africa, it is essential that there are ethical and legal governance structures in place to oversee the operation of these biobanks. Such governance is essential to ensuring that donors are protected, that cultural perspectives are respected and that researchers have a ready availability of ethically sourced biological samples.

**Methods:**

A literature review of all legislation, regulations, guidelines and standard operating procedures on informed consent, confidentiality and the transfer of biological samples amongst countries in Sub-Saharan Africa was conducted. In addition, an examination of the websites of departments of health and national ethics committees was performed. Researchers and research ethics scholars in the field in various African countries were contacted for assistance. A literature review of all studies examining participants views on issues related to biobanking in Africa was carried out and five separate studies were found.

**Results:**

It was found that biobanking guidelines differ substantially across Sub-Saharan Africa regarding biobanking and often conflicted across borders. This has the potential to negatively impact collaboration. Furthermore, the guidelines in place often do not recognise the ethical difficulties arising from the transfer of biological samples and are unsuitable to regulate biobanks. Additionally, there is insufficient research into the views of research participants and stakeholders on the use of biological /samples.

**Conclusion:**

Collaboration is necessary to ensure the success of biobanking projects in Africa. To achieve this, there should be some harmonization of guidelines across Africa which would aid in transferring biological samples across borders. These guidelines should reflect the unique ethical issues arising out of the storage and secondary uses of biological samples. Finally, further research into the views of research participants is necessary. Such studies should aid in the drafting of any new harmonization guidelines.

## Background

For decades, research has flourished in Africa and large volumes of biological samples and data have been transferred to developed countries for storage in biobanks and uncertain secondary use. In June 2010, the National Institutes of Health (NIH) and the Wellcome Trust launched a joint project called “Human Heredity and Health in Africa” (H3Africa) [1]. This project aims to identify the genetic and environmental factors that contribute to common diseases in Africa which will ultimately improve the health of African populations. Central to this project is the establishment of biobanks in Africa which will store and make available DNA, tissue samples and medical information to researchers. This will not only improve the infrastructure and promote research in Africa, but may also lead to increased collaborations both within Africa and across the world [2].

Once operational, the biobanks must ensure that these materials and data are made available “in a manner consistent with national legislation and ethical approvals” [3]. The H3Africa’s High-Level Principles on Ethics, Governance and Resource Sharing also provides guidance on informed consent, confidentiality, the sharing of research samples, data and protocols, providing feedback to research participants, intellectual property rights and community engagement [3]. While this project should build research capacity within Africa many jurisdictions within Africa currently do not have national legislation or guidelines on the use of stored biological samples. Furthermore as these principles recommend a new approach to many long standing ethical principles such as informed consent, they may be in conflict with existing national guidance [4]. These difficulties are further compounded by the lack of research on the perceptions of African people on the use of their stored biological data.

The H3Africa project is not the first initiative to establish biobanks in Africa and a number of local and national biobanks have emerged. The Africa Centre in South Africa has built up an extensive collection of biological samples over the past decade [5]. and the Gambia was the first African country to establish a national DNA bank [6]. However, these biobanks may have emerged without formal legal or ethical oversight. Where guidance is in existence, disparities exist in relation to informed consent and export and import requirements. Additionally, many institutions may be storing samples but they may not be formal biobanks. As the H3Africa project intends to increase the number of biobanks across Africa, there is a need to ensure that there are harmonious legal and ethical guidelines on the storage of biological samples across the African continent. While it is unlikely that there will be agreement on the establishment of one central biobank in Africa [2]. efforts should be made to ensure that there is uniformity of governance of biobanks throughout Africa. This will enable easy transfer of biological samples throughout the continent and ultimately encourage collaboration.

This growth in biobanks is echoed throughout the rest of the world where a number of national (e.g. http://www.decode.com, http://www.genomics.gov) and local biobanks have been established in response to a growing need for ready access of biological samples [7]. Despite the emergence of these national biobanks, many large scale research projects may need biological samples from multiple biobanks and, as such, there should be basic quality and documentation standards across all biobanks to ensure ease of transfer [8]. As a result, there has been the emergence of groups such as the Biobank and Biomolecular Resources Research Infrastructure (BBMRI) which has as one of its aims to create a legal and financial framework for a pan-European biobank infrastructure [9] and the P3G (Public Population Project in Genomics) Consortium which has members throughout the world [8]. Despite these groups, difficulties in international collaboration have emerged where there are differences in guidelines [10].

## Methods

Of concern, unlike international counterparts [11], there is a paucity of studies on the opinions of research participants in Africa on the ethical issues raised by biobanking [12] with the only studies focusing on ethical issues encountered by Ethical Review Committees (ERCs) in Kenya [13], participants in a Nigerian study on storage and reuse [14], two South African studies in which participants were asked their views on storage and reuse [15,16], seeking consent for genetic research in Ghana [17] and the views of Ugandans on stored biological samples [18]. Thus to ensure that good governance of biobanks in Africa complements the H3Africa project, future research is required to assess the opinions of research participants in addition to harmonious legal and ethical guidelines throughout Africa. In particular, focus must be given to consent procedures, confidentially as well as the importing and exporting of biological samples. Although other issues such as the return of research results and research and the participation of children in biobanking are also hotly debated, the three issues that follow will be the focus of discussion in this paper.

## Results

### Informed consent

The need to adequately obtain informed consent prior to research involving human participants is a fundamental ethical principle [19]. Its origin is rooted in the Nuremberg Trials and is founded on the basis that individuals have the right to decide what is done with their bodies [19] and that any risk associated with research must be voluntarily accepted [10]. The informed consent process has been described as the ‘moral contract’ between the participant and researcher as it sets out the framework though which their biological samples may be used [14]. Thus, the consent document should outline the purpose for which their biological samples will be used and consent should be sought on this basis alone [20].

The difficulty with biobanks is that the potential secondary uses of the samples may not be known at the time of consent. Thus, to conform to the principle of informed consent, participants must be re-contacted to obtain their re-consent [14] which may potentially drastically reduce the number of available samples [10]. Due to the onerous and time-consuming task that this presents, it has been suggested that the traditional understanding of informed consent must be re-examined in light of biobanking or risk negatively impacting population genetic research [21]. As the use of a biological sample contains little physical risk for the participant [10] and biobanking potentially has a potential considerable social value [22], it has been argued that there should be a move away from a strict informed consent doctrine in biobanking with broad consent and tiered consent proposed as two options.

Broad consent occurs when a donor consents to the use of their biological samples for future, unspecified research. This simplifies the consent process as it negates the need to re-contact the donor and has also received the support of the World Health Organisation (WHO) [23]. If the risks and information provided in the original consent are common to several studies, then arguably the original consent may be sufficiently informed [10]. However if no similarities exist between the studies, the consent cannot be informed and is thus contrary to the Declaration of Helsinki. While arguably the level of information a person must be given before their consent can be considered informed is uncertain, a donor cannot possibly make an autonomous decision based on little or no information. While admittedly there is a need to re-examine the “one study/one informed consent” paradigm [24] in the context of biobanking, Caulfield et al. have argued that as broad consent is quite vague, it is too general to have any legal weight [21].

Tiered consent provides donors with a range of consent options; they may opt for broad consent, opt to be recontacted before the sample is used, or opt for a REC to consent on their behalf [22]. Such a model conforms to the Organisation for Economic Development (OECD) guidelines on Human Biobanks and Genetic Databases which requires a review process where samples are to be used in a manner not anticipated in the original consent form [25]. While such a model does not do away with the difficulties of potentially contacting all donors to obtain their reconsent or address all the difficulties that broad consent raises, by presenting a donor with a range of options from which they can choose, strikes a balance between advancing the interests of science and respecting the autonomy of participants [26].

It would also appear that there is support in Africa for these new models of consent. In a study examining attitudes to biobanking in Nigeria, Igbe *et al.* reported that over half the participants were in favour of broad consent, a quarter supported some form of restricted consent and the remaining quarter supported tiered consent. Yet, even amongst those who supported broad consent, there was concern that there should be some additional control before the samples are used in research. For example, the approval of the Nigerian Federal Ministry of Health must be sought or safeguards be put in place to prevent the unethical use of the samples [14]. Similarly, Wendler *et al.* have found that a study in Uganda indicated a high level of support for sample reuse based on future institutional review board (IRB) approval [18]. These findings were echoed in a South African study which indicated, that while participants were willing for their sample to be used in future research, they would attach conditions to its reuse [15].

While these are small scale studies and the findings should not be taken as representing the views of all of Africa, they do indicate that there is a willingness to go beyond the traditional informed consent doctrine in the context of biobanking. However the apparent support of broad consent should not be taken at face value as closer examination of the responses found that participants would want some checks put in place to ensure that the research conforms to ethical norms. Under a tiered consent model, the donor could opt that research ethics committee (REC) approval is obtained and this would be in accordance with the Declaration of Helsinki which states that reconsent can only be waived upon approval of a REC. It would then be the role of the REC to determine whether the research can proceed or whether reconsent is necessary. If donors wish that there is some control over the use of their samples, this additional check by the REC may prove necessary. A review of protocols submitted to two Ethics Review Committees in Kenya found that less than half who requested the use of samples for their research contacted the donors [13]. Yet the findings of Moodley *et al.* in a small South African study have suggested that some have reservations about RECs consenting on their behalf, with 50% of participants indicating that they would not want a REC to consent on their behalf [16]. If this reservation towards RECs is Africa-wide, a tiered consent model could address this issue as the donors can opt that they be recontacted or appoint some other body to consent on their behalf.

In contrast to these findings, regulations in Africa tend to focus on the traditional informed consent doctrine with a role for the REC in secondary use. The South African National Health Act 2003 requires informed consent before a biological sample is donated for research. While the legislation is silent as to reuse, the Ethics in Health Research Guidelines state that each research institution should draft guidelines as to when reconsent is required and when a waiver of consent may be obtained [27]. National guidelines on this issue would be preferable as otherwise institutions may apply differing ethical principles.

In Botswana, the Standard Operating Procedures for Biomedical Research requires donors to be given the option of deciding whether their samples should be stored for future use and the Health Research and Development Committee (HRDC) must approve any protocol which seeks to reuse the tissue samples [28]. The Gambia developed its guidelines in 2001 in response to the establishment of its National DNA bank. The guidelines do not require the consent of the donor if the samples have been anonymised and unlinked, but rather the consent of the Gambia Government/MRC Laboratories Joint Ethics Committee (the Ethics Committee). Where the samples have not been anonymised, in principle informed consent should be required but this may be waived by the Ethics Committee [29].

By contrast, in Uganda, a consent form separate from the form enrolling a participant in a study is required to obtain and store a biological sample. This form must detail where the sample will be stored, the purpose of the study, how their confidentiality will be protected and the conditions surrounding the future use of the sample. At this point donors are given the option of deciding whether their samples may be stored for future use [30]. Sudan’s guidelines similarly require the consent document to discuss whether there is the possibility of the potential secondary use of the samples, whether that use will be limited, the type of research which may be carried out on the samples and the conditions under which the donor may be contacted to authorise reuse [31].

The recently passed Zambian National Health 2013 provides for the establishment of biobanks but states that biological material can only be collected for the manner in which it was indicated in the research protocol. It is currently unclear whether an exception will be made for future use for research and under what conditions the consent can be waived. Other jurisdictions fail to give guidance. Kenya’s National Guidelines are silent on the issue while both Ethiopia [32] and Malawi [33] discuss the procedures for importing and exporting samples but give no guidance as to the consent required for future reuse.

### Confidentiality

Central to good governance of biobanks is the requirement that the confidentiality of donors be maintained. This not only includes ensuring that access to the data is limited so that donors cannot be identified by certain third parties, but also that any results are kept confidential to ensure that there is no risk of discrimination or stigmatisation. The risk of stigmatisation and discrimination is a real concern in genetic research and in April 2010, Arizona State University agreed to pay $700,000 to members of the Havasupai Indian tribe to settle claims that the improper use of the tribe’s blood samples stigatimised the tribe [26].

The UNESCO Declaration on Human Genetic Data requires states to protect the privacy of an individual and the confidentiality of genetic information which is linked to an identifiable person, family or group. To achieve this, samples can be either anonymised whereby all indentifying data has been removed or coded [34]. The codes may be both double or single coded and the sample can only be identified by breaking the unique codes of the sample [7]. To fully understand their involvement with the biobank, donors must know whether their sample will be anonymised [25]. However developments in medical informatics and bioinformatics have demonstrated that privacy and confidentiality can no longer be guaranteed [4] and that with genetic samples, re-identification is possible [35]. DNA in itself is an identifier [36], thus no matter how anonymised data is, there is the potential that it may be identified [37].

A further problem with anonymised samples is that it can limit the usefulness of the sample. The value of biobanks is that it combines health and genetic data together [38] and the health data is as important as the genetic information. While anonymonising the sample protects the donors identity and it is thus “ethically and legally expedient by avoiding the possibility of re-identifiability” [39], this limits the sample’s usefulness as no other data can be added to it [12]. On the other hand, if the sample is coded, the clinician can send ongoing information via a third party who is the code key holder and the researcher can send information to the clinician in the same manner. Furthermore coding allows the identification of the sample and enables the donor to withdraw at any time and also to be recontacted for secondary use [7,40]. Anonymised samples cannot be re-identified, preventing donors from withdrawing their sample. Thus it has been argued that there should be a strong ethical presumption in favour of coding as donors can retain some control over their samples [41].

Internationally there is growing move away from anonymonity with the WHO guidelines cautioning that anonymity may be breached [42]. The CIOMS guidelines also acknowledge that there are instances in which a sample may not be anonymised but in such circumstances, they must be coded and this must be explained to the donor during the consent process. In the US, IRB approval for the use of coded samples in not necessary provided the researchers cannot readily ascertain the identity of the donor and the specimens were not collected for the currently proposed research project [43]. Thus provided confidentiality can be assured, coding appears to be a suitable alternative.

In the limited African studies on this issue, confidentiality has been raised as a concern. Fear of discrimination or stigmatisation due to results has been expressed [14] and it has been reported that research participants do not always think that researchers will always act in their best interests [15]. These are real fears particularly as genetic discrimination may affect several generations of genetic relatives [21]. Thus it is essential that biobanks have policies concerning confidentiality in place and in this way maintain the trust of the public.

Despite the growing trend towards coding, it is only the Gambia that directly addresses the issue. The guidelines state that, where possible, samples should be anonymonised and unlinked. However they also state that confidentiality can be protected through a coding system with the codes to be held by the Head of Human Genetics, the Director of the MRC, and the Gambian Government [29]. Despite coding appearing to be a pragmatic response to ensuring confidentiality in genetic research as it balances the privacy of the donor with progress of science, no other jurisdiction addresses this issue [40].

In contrast, the South African National Health Act 2003 requires the genetic material to be kept confidential and the National Guidelines requires the REC overseeing the establishment of a biobank to ensure that provisions are made to ensure the confidentiality of the data [27]. Yet both are silent as to whether the sample should be anonymised or coded. Sudan’s national guidelines do go further and state that biological samples cannot be used unless it is demonstrated to the REC that the patient’s “privacy and confidentiality or anonymity are assured” but makes no explicit mention to coding and whether this is preferred over anonymity [31]. The rest of the African continent appears to be silent on the issue.

### Importing and exporting samples

Large scale population studies will require a large amount of samples thus it may be necessary to transfer biological samples across borders [22]. However, access and use of materials is a charged issue, particularly in developing countries where many communities are reluctant to permit foreign researchers access to human tissues due to past exploitation of vulnerable groups [44]. For example, in 2007 Indonesia refused to share its H5N1 samples without a legally binding agreement which addresses benefit arrangements and intellectual property rights [45]. Thus the transfer of biological samples raise issues such as ownership of the sample and ownership of any future intellectual property rights arising out of future use of the sample. More fundamentally, donors themselves may object to the exporting of their samples, preferring them to be stored in their home country in line with the legal and ethical requirements there. Thus considering all the issues previously discussed in relation to recontacting a donor, should this consent be required prior to the exportation of any sample?

Few studies have explored participant views on the export of samples. A Ugandan study in which participants expressed a willingness to share their samples with researchers in the US and UK [18]. In contrast, a recent South African study found that participants were concerned about sharing their samples with other African countries as well as with developed countries like the UK and the US [15]. There is a clear need for further research in this area and it is unsurprising that there have been previous calls for research into the extent to which tissue exportation is consented to in Africa and the extent to which communities understand the rationale for tissue exportation [46]. This is particularly pertinent in light of a study which indicated that the participants were convinced that the researchers were going to sell their samples abroad. Although a deep mistrust of the researchers was evidenced in this study, it does serve to illustrate the lack of trust which may exist and a belief that researchers are going to benefit through the sale of their samples [47].

Despite these concerns, the limited guidance does not address the issue of donor consent. In South Africa, a biological sample may not be imported or exported without a permit issued by the Director-General. An export permit will not be issued unless it is proven that the biological sample was donated under the terms of the Act and that the sample will be used within the terms of the Act. This would indicate that informed consent is required, but the legislation is not only silent on the issue as to whether consent is required for reuse of the sample, but there is no requirement to seek the consent of the sample donor prior to the issuance of an export permit. The National Guidelines provide no further guidance but simply state that the REC should review and approve the protocol under which data and samples may be shared [27]. Due to the need to seek REC approval, it would appear that donor consent is not necessary and REC approval will suffice. Yet considering Moodley *et al*’s study which indicated some reluctant to the transfer of biological samples, this is an issue which must be addressed in South Africa and likely, throughout the African continent.

Surprisingly, the Act does not require the completion of a Material Transfer Agreement (MTA) before a permit is issued. In contrast, the Nigerian ethical guidelines require a MTA detailing the use of the sample, its location, the duration of the storage and the transfer and termination of the use of the sample. The guidelines further state that a MTA must also be signed by all parties to the research, but there is no requirement that the donor’s consent be obtained prior to the exportation of the sample. The Zambian National Research Act 2013 requires a MTA prior to the transfer of any samples and this MTA must describe the ownership of the materials and any intellectual property rights arising out of the materials. Botswana similarly requires a MTA prior to the exchange of any biological samples. However there is no further guidance on the procedures to be followed except that clearance shall be granted by the HRDC.

Uganda appears to put some limits on the transfer of samples abroad to situations when the country does not have the capacity to perform the necessary research. In such circumstances the transfer must be approved by the Uganda National Council for Science and Technology, a MTA must be signed and the applicant must be a legal resident of Uganda or affiliated to a legally recognised institution in Uganda. However there are no requirements that the consent of the donor be obtained prior to the removal of the sample. Similarly the Ethiopian guidelines state that a sample may be exported if it requires research which is not currently underway in Ethiopia or need to be rechecked by a skilled worker and a MTA must be signed [32]. Despite the legal and ethical issues which the transfer of biological samples entails, there does not appear to be guidance on this matter elsewhere in Africa (Table 1).

**Table 1:** Table of sources

**Organisation/Country**	**Code/Legislation/Guideline**	**Topics covered**
OECD	OECD Guidelines on Human Biobanks and Genetic Research Databases (2009)	Privacy, confidentiality, informed consent, re-contact, re-consent
World Medical Association	Declaration of Helsinki	Informed consent, re-contact, re-consent
South Africa	National Health Act 2003	Informed consent, confidentiality, exporting/importing samples
	Department of Health: Ethics in Health Research: Principle, Structures and processes	Secondary use of samples, informed consent, re-contact, re-consent, waiving of consent, confidentiality
Nigeria	National Health Research Ethics Committee of Nigeria (NHREC) National Code of Health Research Ethics 2007	Export/Importing of samples, MTAs
Kenya	National Council for Science and Technology Guidelines for Ethical Conduct of Biomedical Research Involving Human Subjects in Kenya (2004)	Informed consent, re-contact, re-consent
Botswana	Ministry of Health Standard Operating Procedures for Review of Biomedical and Bio-behavioural research in Botswana	Informed consent, export/importing of samples/MTAs
The Gambia	Guidelines of the National DNA bank, The Gambia (2001)	Confidentiality, coding, anonymisation, informed consent, re-contact, re-consent
Uganda	Uganda National Council for Science and Technology National Guidelines for Research Involving Humans as Research Participants 2007	Informed consent, confidentiality, future use of samples, export/importing of samples, MTAs
Zambia	The National Research Act 2013	Informed consent, export/importing of samples, MTAs
Ethiopia	Ethiopian Science and Technology Commission National Health Science and Technology Council, National Research Ethics Review Guideline 2005	Export/importing of samples, MTAs
Malawi	National Health Sciences Research Committee, Policy Requirements, Procedures and Guidelines for the Conduct and Review of Human Genetic Research in Malawi (Sept 2012)	Export/importing of samples, MTAs
Tanzania	Human DNA Regulation Act 2009	Informed consent
Sudan	National Guidelines for Ethical Conduct of Research Involving Human Subjects (2008)	Informed consent, confidentiality, privacy, secondary use of samples

## Discussion

Medical progress should not result in an erosion of ethical principles. The basic idea of informed consent, emanating from the Nuremberg trials, is that any risk associated with a research protocol must be accepted on a voluntary basis. The need for an informed and voluntary assumption of risk seems to preclude broad consent and future consent. However, if the risk of harm from future biomedical research is low and sufficiently well controlled, if the participant voluntarily accepts this level of risk, and if there is a mechanism for withdrawal, then there is no reason why broad consent and future consent to such studies should not be acceptable.

The issue regarding consent in biobanking is raging worldwide and no real consensus has yet emerged on this issue [48]. It is clear that while consent is important and should be an essential feature of the biobanking process, the notion of informed consent being a core ethical principle may need to be re-examined for biobanking. While this article will not recommend whether specific consent, tiered consent or broad consent should be adopted throughout the African continent, what is important is that there is a consistent approach to consent. Currently, South Africa adheres to the traditional informed consent doctrine which has been demonstrated to be problematic in the biobanking context, while Uganda has detailed procedures which must be satisfied prior to the storage of a sample. Problems are likely to arise when samples are exported throughout the continent. Can a country with strict consent provisions import or export samples to those with no guidelines? Uniform or similar consent provisions would ensure that problems like this do not occur and risk damaging collaborations throughout Africa. Furthermore there is a need for future studies to determine whether donors favour informed consent, broad consent or tiered consent. Regulations on biobanking should reflect the findings of these studies to ensure that biobanking research has the support of the public.

However, regulations should also have a degree of flexibility. In the aftermath of Hurricane Sandy, it was reported that due to power outages, freezers and incubators in which biological samples were stored in New York stopped working. To save the samples, blocks of ice had to be manually transported up 18 floors and over the following days the samples were transported to different laboratories [49]. As Africa frequently experiences power outages and power surges, measures such as this may be required to be taken to protect the samples and ensure that they can be continued to be used. Such issues must be explained when informed consent is sought.

The greatest risk to research participants in biobanking rests on access to information and confidentiality [50]. Due to the potential identification from genetic material, it is important that biobanks have policies in place to ensure that confidentiality is maintained. If the identity of donors cannot be kept confidential, the arguments in favour of any form of broad consent weaken. While an anonymonised sample is the best means of ensuring privacy this limits the potential for a donor to withdraw their sample. Thus the coding of samples is perhaps the best policy to have in place. Irrespective of which option is chosen, it is crucial that standardised policies are put in place throughout Africa. Public trust in the biobank can only be maintained with policies detailing how confidentiality will be maintained. Currently there is a lack of regulations on the maintenance of donor’s privacy in Africa and this must be urgently addressed.

A further issue which needs closer examination is the transfer of biological samples throughout Africa. It is currently unclear whether the ethical standards of the country exporting the sample must meet that of the host country. To ensure that all samples have been ethically sourced, the UK Human Tissue Authority’s Code of Practice recommends that the importer put in place SOPs which clearly sets out how informed consent was obtained and how the information will be treated as confidential. Furthermore the material must be sourced in accordance with the ethical and legal review standards of England, Wales and Northern Ireland [51]. Similar provisions should be put in place in Africa as it is difficult to ethically justify the importation of tissues from countries which were not in accordance with the legal and ethical rules of that country. The legal and ethical principles should apply irrespective of the origin of the samples.

These are issues which must be outlined in the guidelines and be a requirement prior to the issuance of an export permit. Importantly, due to the intellectual property issues which can arise in the transfer of samples, it is important that a MTA be agreed between all parties. MTAs are important in the transfer of samples as they clearly issues such as the secondary use of the samples, commercial use of the samples and any liability associated with the samples [52]. This will avoid any potential future conflicts.

## Conclusion

Biobanking has great potential to build research capacity in Africa. However there is currently a lack of research into the attitudes of research participants towards the storage and secondary use of tissues in Africa. Due to the new issues that biobanking raises in relation to informed consent and confidentiality, further research on both of these issues is necessary. Furthermore, new regulations are necessary to ensure that basic legal and ethical principles are adhered to and these regulations must reflect the changes to basic ethical principles such as informed consent and privacy. Not only should these regulations be introduced at a national level, but they must be done so in accordance with other African nations. This may pose challenges to some countries involved in the H3Africa initiative who must introduce regulations concerning these issues which reflect local cultural values, while also cognisant of the wider guidelines underpinning the H3Africa project. Previous studies may guide such countries in introducing new or amended regulations, but new studies which reflect the particular issues that the H3Africa project brings are necessary before such regulations are introduced. Such an approach should help in the introduction of a harmonious legal regime throughout Africa which will ensure an ease of transfer of samples throughout Africa. Indeed a harmonious legislation throughout the world would be preferable as this would increase worldwide sample sharing and improve collaboration. However, as the H3Africa project is specifically focused on Africa, it is necessary that particular focus is given to ensuring a harmonious regime in Africa first as this will ultimately benefit collaboration and the H3Africa initiative.

## Competing interests

The authors declare that they have no competing interests.

## Authors’ contributions

CS and KM jointly conceptualised the idea for this paper. CS conducted background research, reviewed legislation that is currently available, and perused all available literature. She communicated with various researchers and scholars in the field in Africa and collated data from various African countries, to the extent possible. CS drafted the first version of the paper and implemented changes in subsequent drafts after discussion with KM. In general, KM supervised the project, sourced literature on the opic and provided a list of researchers and research scholars in Africa. KM revised drafts of the paper and approved the final version for submission for review. Both authors read and approved the final manuscript.

## Pre-publication history

The pre-publication history for this paper can be accessed here:

http://www.biomedcentral.com/1472-6939/14/35/prepub
